# Supplementary material for: Diffuse idiopathic skeletal hyperostosis was the specific risk factors of methicillin-susceptible Staphylococcus aureus spine infection: a retrospective study in a single center
Source: PeerJ. 2024 Nov 26;12:e18432. doi: 10.7717/peerj.18432 (PMC11606330; doi:10.7717/peerj.18432)
Supplement: Supplemental Information 2 [file peerj-12-18432-s002.pdf]

Supplemental file 1. MSSA bacteremia patients data set: age, sex, diagnosis, past history of diabetes (DM), steroid use, CRP day1, WBC day1, CRP day3, WBC day3, CRP rate of day1 / day3 (CRP rate), WBC rate of day1 / day3 (WBC rate), past history. Group means spine infection groups as group A, the other organ infection as group B and implant infection as group C. Outcome included the mortality rate and spine infection included modified Frankel classification. Infection level means spine infection site. CT data analysis data were Diffuse idiopathic skeletal hyperostosis (DISH), osteoarthritis (OA), bridging, scoliosis in whole spine.

Age	Sex	Diagnosis	DM	Steroid use	CRP day1	WBC day1	CRP day3	WBC day3	CRP rate	WBC rate	Past history	Group	Outcome	Infection Level	DISH	OA	Bridging	Scoliosis
70	M	Facet infection	-	-	20.7	14,400	16.8	9,500	81.1	66.0	HT, HL, DISH (T-spine)	A	E	L4/5 Left facet	+	+	+	-
18	M	Facet infection	-	-	8.3	13,300	5.8	8,100	69.8	60.9	-	A	E	L2/3 Left facet	-	+	-	+
50	M	Facet infection	-	-	27.1	11,500	23.4	10,300	86.3	89.6	Atopic dermatitis	A	D3	L4/5 Right facet	-	+	-	+
79	F	Discitis	-	-	30.7	26,000	32.1	15,900	104.4	61.2	RA, HT, DISH (T-spine)	A	Death	T9	+	+	+	-
66	M	Facet infection	-	+	22.7	3,200	11.3	3,300	49.8	103.1	Nephrotic syndrome, Stroke	A	E	L5/S1 Right facet	-	+	-	+
89	F	Discitis	-	-	11.0	10,400	8.0	8,100	72.7	77.9	Urinary tract cancer	A	A	L3/4	-	+	+	+
83	F	Discitis	-	-	23.0	19,500	17.3	15,800	75.1	81.0	AR, MR	A	B	T12/L1, L4/5	-	+	+	+
89	M	Discitis	-	-	26.3	41,200	23.6	37,400	89.9	90.8	AP, HT, CKD, Stroke, LCS, KOA, DISH (T-spine)	A	Death	L3/4	+	+	+	+
57	M	Discitis	-	-	7.4	17,400	26.8	11,400	361.8	65.5	CKD, DISH (T-spine)	A	E	L2/3	+	+	+	-
70	M	Discitis	-	-	9.0	15,400	20.3	7,500	225.1	48.7	T12,L1 burst fracture, DISH (T-spine)	A	E	L4/5/S1	+	+	+	+
88	M	Discitis	-	-	25.1	24,300	18.8	26,900	74.9	110.7	-	A	Death	T9/10	-	+	+	+
58	F	Discitis	+	-	7.0	19,300	6.6	13,000	94.2	67.4	HHS	A	E	L5/S1	-	+	+	+
75	M	Facet infection	-	-	27.4	10,800	16.2	7,900	59.2	73.1	Coronic cancer, Prostate cancer, Stroke, Af	A	D3	L3/4/5/S1 Right facet	-	+	+	+
79	M	Discitis	-	-	7.5	15,400	14.9	6,700	197.9	43.5	T12 burst fracture, DISH (T-spine)	A	B	T12/L1/2	+	+	+	-
84	F	Bacteremia	-	-	3.7	8,000	1.2	2,600	32.1	32.5	Dialysis, Encephalorrhagy, Gallstone	B			-		-	+
53	M	Bacteremia	-	-	11.0	21,000	21.8	15,900	198.0	75.7	HT, HOA	B			-		+	-
75	F	Aortitis	+	+	14.9	26,400	11.1	23,100	74.6	87.5	Athma, Coronic cancer, Thyroid cancer, Hysteromyoma, HT,	B	Death		-		-	-
76	M	Pneumoniae	+	+	3.4	4,500	7.7	7,200	225.2	160.0	DM, BPH, Dialysis, CHF	B			-		-	-
66	M	Cellulitis	-	-	23.7	9,000	16.5	15,600	69.6	173.3	Colonic cancer, HT	B			-		+	+
31	M	Bacteremia	-	-	13.6	16,300	4.3	5,200	32.0	31.9		B			-		-	+
88	F	Bacteremia	-	+	8.8	11,500	6.0	9,000	68.4	78.3	BPH, Esophageal cancer	B			-		+	-
91	F	Abcess	-	-	14.1	7,500	36.7	6,200	261.1	82.7	Stroke,Diverticula, Parkinson's desease	B	Death	Knee	-		+	+
62	M	Cellulitis	-	-	19.1	8,700	11.7	9,000	61.1	103.4	SSS(PM), HT, Parkinson's desease, Depression	B		Foot	-		+	-
94	F	Bacteremia	-		26.2	16,600	26.0	14,400	99.4	86.7	OMI, BA, Left pneumonectomy (TB)	B	Death		-		+	-
75	M	Bacteremia	-	+	2.4	8,100	1.7	8,800	73.0	108.6	HT, Encephalorrhagy	B			-		-	-
76	M	Abcess	+	-	38.0	27,000	29.5	25,400	77.5	94.1	DM	B		Perianal abcess	-		+	+
95	F	Bacteremia	+	-	17.0	19,600	12.5	10,300	73.5	52.6	CHF(CABG), BPH, Ureterolithiasis, DM	B	Death		-		+	+
88	F	Cellulitis	-	+	14.3	14,000	19.3	15,000	134.6	107.1	Colonic cancer, IP	B	Death	Foot	-		+	+
90	F	IE	-	-	14.3	11,100	16.4	16,000	115.2	144.1	Stomac cancer	B			-		+	+
42	M	Bacteremia	+	-	39.5	22,500	11.9	21,400	30.3	95.1	DM, Alchoholic	B			-		-	-
85	F	Bacteremia	+	-	8.3	23,300	6.6	22,800	79.3	97.9	Dimentia, Schizophrenia, DM, Parkinson desease	B			-		+	+
72	M	Abcess	-	-	13.5	5,600	8.9	17,300	66.2	308.9	Af, DM, Dimentia, HT	B	Death	Head	-		-	-
70	M	Ileus	+	-	28.3	10,400	37.2	17,800	131.5	171.2	Stroke, Dialysis, DISH (Whole spine)	B			+		+	+
65	M	Abcess	+	-	30.5	10,200	36.9	11,200	121.1	109.8	Stroke, CHF(CABG)	B		Right forefinger	-		+	-
84	M	IE	-	-	11.3	16,900	8.6	2,800	76.4	16.6	Stroke, AP, MR, CHF, COPD	B			-		+	+
33	F	IE	-	-	10.3	20,900	19.9	20,400	193.5	97.6	-	B			-		-	+
79	M	Pneumoniae	+	-	5.0	9,800	7.7	7,200	154.6	73.5	SAH, DM, HT, HL, AP, Dialysis, PM	B			-		+	-
89	M	Bacteremia	+	-	10.3	14,500	10.2	16,700	98.8	115.2	ASO, CHF, Pacemaker, BPH, DISH (T-spine)	B			+		+	-
87	M	CVC infection	-	+	3.6	11,200	1.7	11,100	47.8	99.1	Stroke, HT, Stomach cancer, DISH (T-spine)	C	Death		+		+	+
89	F	CVC infection	-	-	0.3	2,900	0.3	2,800	93.5	96.6	Dialysis, Htpertension	C			-		-	+
93	M	CVC infection	-	-	21.1	6,000	14.9	8,400	70.9	140.0	Stomach cancer, HT, DISH (T,L-spine)	C			+		+	+
72	F	CVC infection	-	-	18.5	8,480	12.0	8,200	64.9	96.7	Drug rush	C	Death		-		-	+
92	M	Port infection	-	-	8.8	11,500	6.0	9,000	68.4	78.3	BPH, Esophageal cancer	C			-		+	+
79	M	CVC infection	+	-	3.8	13,100	11.1	12,800	292.9	97.7	DM, Stroke,Prostate cancer, anorexia	C			-		+	+
20	M	Port infection	+	-	2.5	10,700	5.0	6,700	200.8	62.6	Alcoholism	C			-		-	-
83	F	CVC infection	+	-	14.4	13,400	22.2	14,200	154.2	106.0	HT, DM, ,HL, pneumoniae	C	Death		-		+	-
85	M	CVC infection	-	-	16.7	26,900	21.2	21,200	126.9	78.8		C			-		+	+
82	F	Port infection	+	-	4.6	18,200	19.1	8,700	419.3	47.8	MI	C			-		-	-
75	M	PVC infection	-	-	22.8	15,000	22.7	14,500	99.3	96.7	-	C	Death		-		+	+
90	M	PM infection	+	-	17.0	19,600	12.5	10,300	73.5	52.6	Dialysis, AP, BPH, Stroke, cholecystitis	C	Death		-		+	-
81	M	CVC infection	-	-	5.4	7,200	4.8	7,500	89.3	104.2	HL	C			-		+	+
89	F	CVC infection	-	-	12.8	7,200	16.0	6,400	125.0	88.9	UTI, Pneumoniae	C	Death		-		-	+
92	F	CVC infection	-	-	4.6	17,700	7.3	7,000	158.2	39.5	Pacemaker, HT, Hashimoto's disease	C			-		+	+
75	M	PVC infection	+	-	10.5	11,100	11.8	12,400	112.9	111.7	Stroke, Cholecystitis	C			-		+	+
62	M	CVC infection	-	-	12.7	9,400	20.4	11,500	160.7	122.3		C			-		+	-
78	F	PVC infection	-	+	4.6	16,400	14.2	5,600	307.2	34.1	Clone desease, Hemophagocytic lymphohistiocytosis	C			-		+	+
87	F	CVC infection	+	-	12.3	15,600	7.9	8,500	64.0	54.5	DM, AP	C			-		+	+
49	M	CVC infection	+	-	3.3	12,900	4.0	8,500	121.0	65.9	Type1 DM	C			-		+	+
87	F	CVC infection	+	-	9.9	12,800	12.1	11,400	122.2	89.1		C			-		+	+

Abbreviation	
AP	Angina pectoris
BA	Bronchial asthma
BPH	Benign prostatic hyperplasia
CHF	Choronic heart failure
CKD	Choronic kidney failure
CVC	Central venus catheter
DISH	Diffuse idiopathic skeletal hyperostosis
DM	Diabetes
HL	Hyper lipidemia
HOA	Hip osteoarthritis
HT	Hyper tension
IE	Infectious endocarditis
IP	Interstitial pneumonia
KOA	Knee osteoarthritis
LCS	Lumber spinal canal stenosis
MI	Myocardial infarction
OA	osteoarthritis
PM	Pacemaker
PVC	Peripheral vein catheter
